# Supplementary figures and images for: Host syndecan-1 promotes listeriosis by inhibiting intravascular neutrophil extracellular traps
Source: PLoS Pathog. 2020 May 26;16(5):e1008497. doi: 10.1371/journal.ppat.1008497 (PMC7274463; doi:10.1371/journal.ppat.1008497)

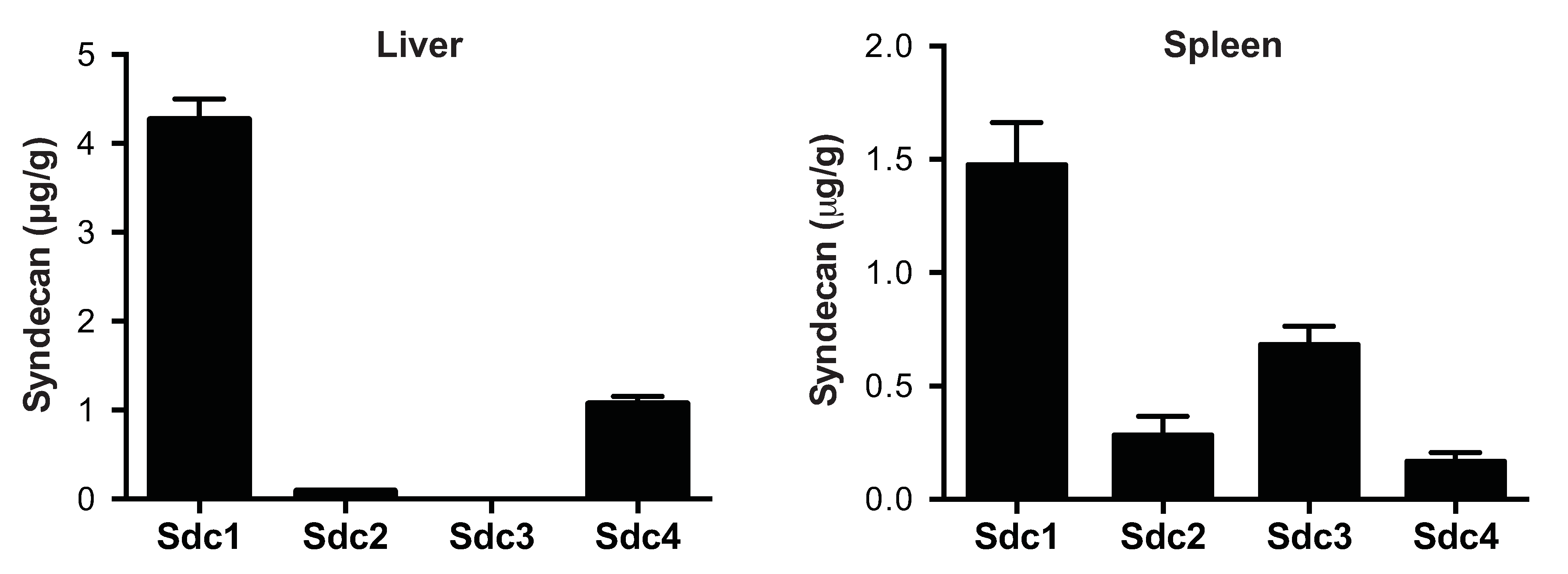

Supplement: S1 Fig — Tissue levels of the 4 syndecans in urea extracts were measured by dot immunoblotting (n = 3). (TIF) [file ppat.1008497.s001.tif]

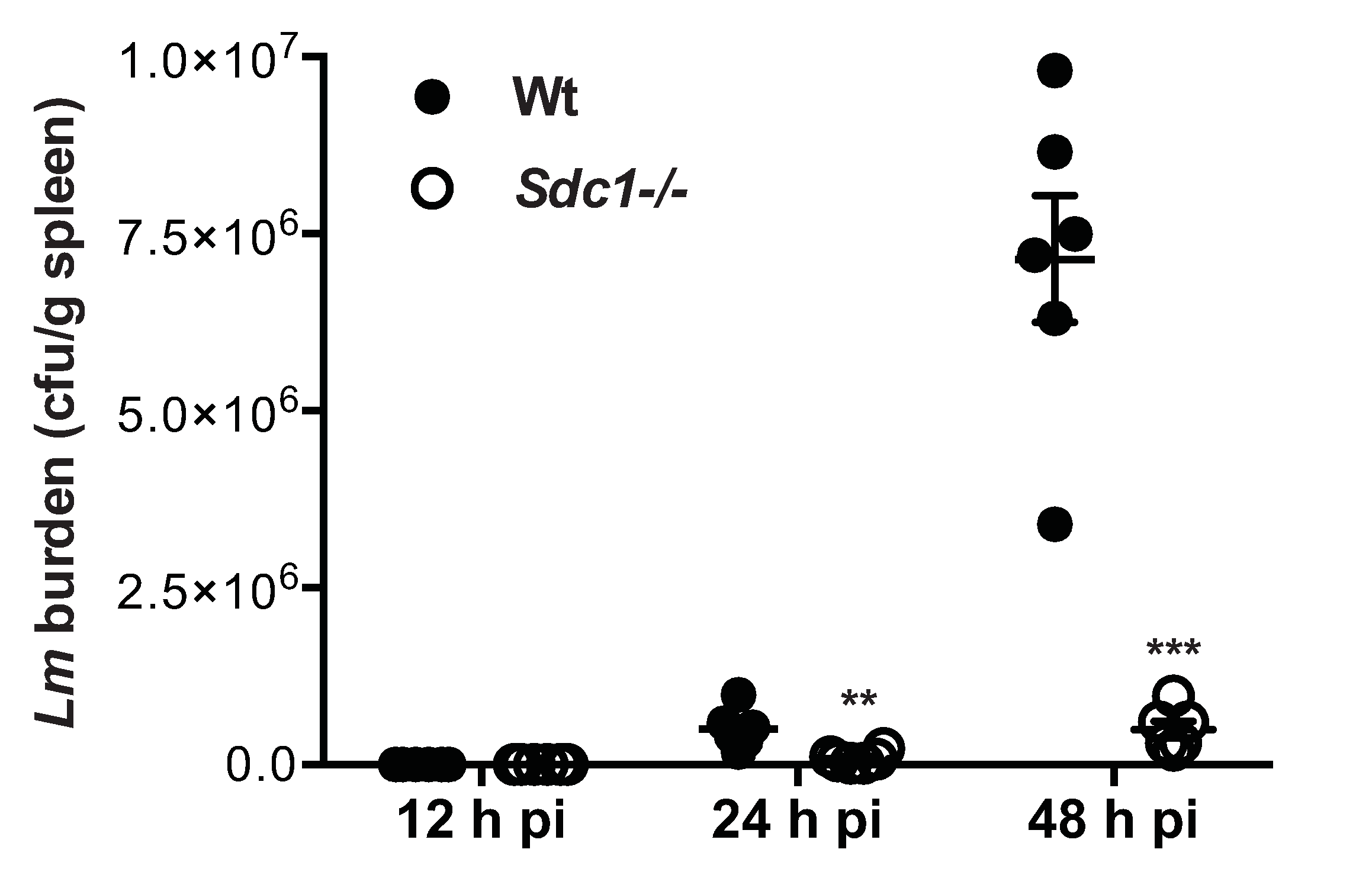

Supplement: S2 Fig — (TIF) [file ppat.1008497.s002.tif]

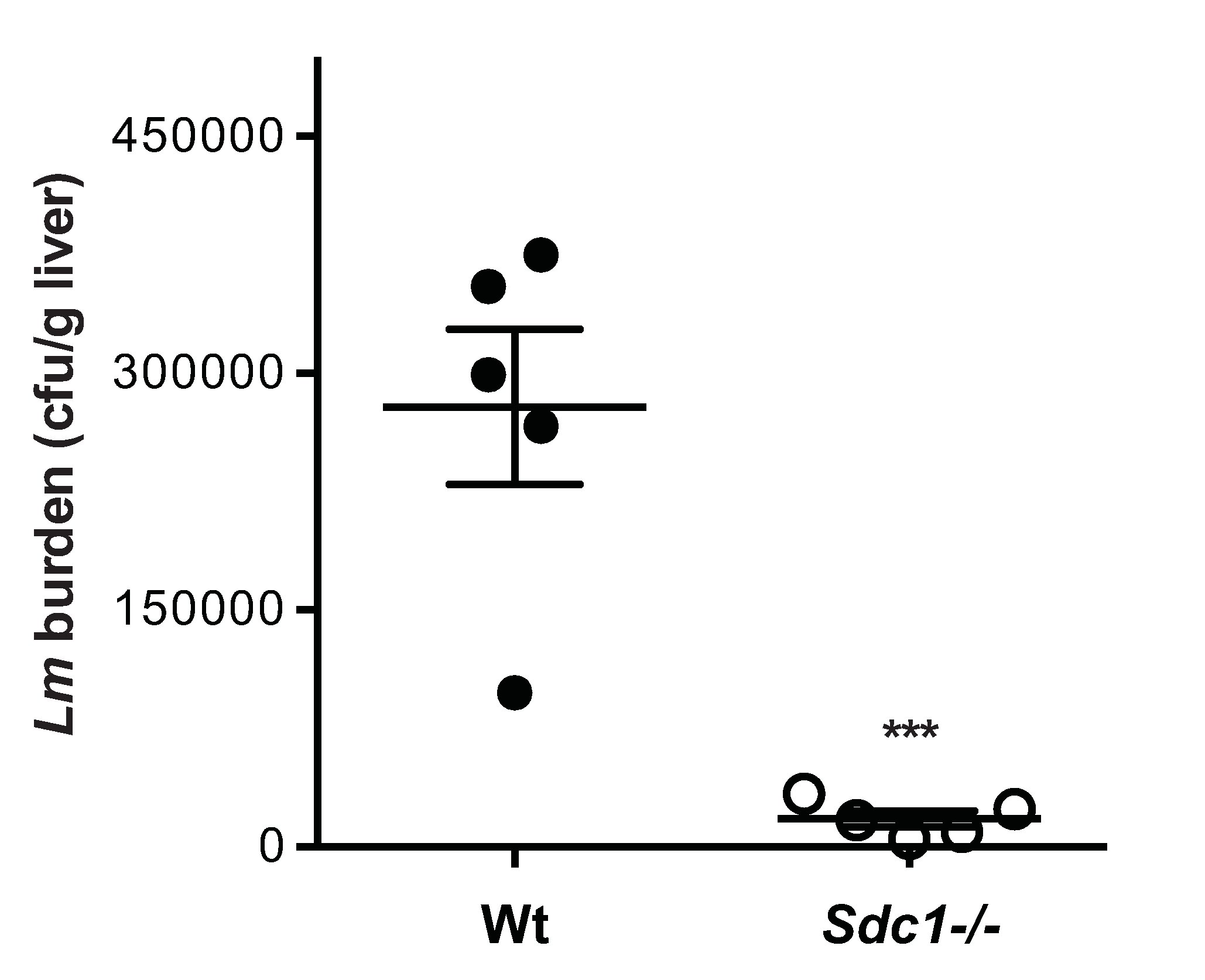

Supplement: S3 Fig — (TIF) [file ppat.1008497.s003.tif]

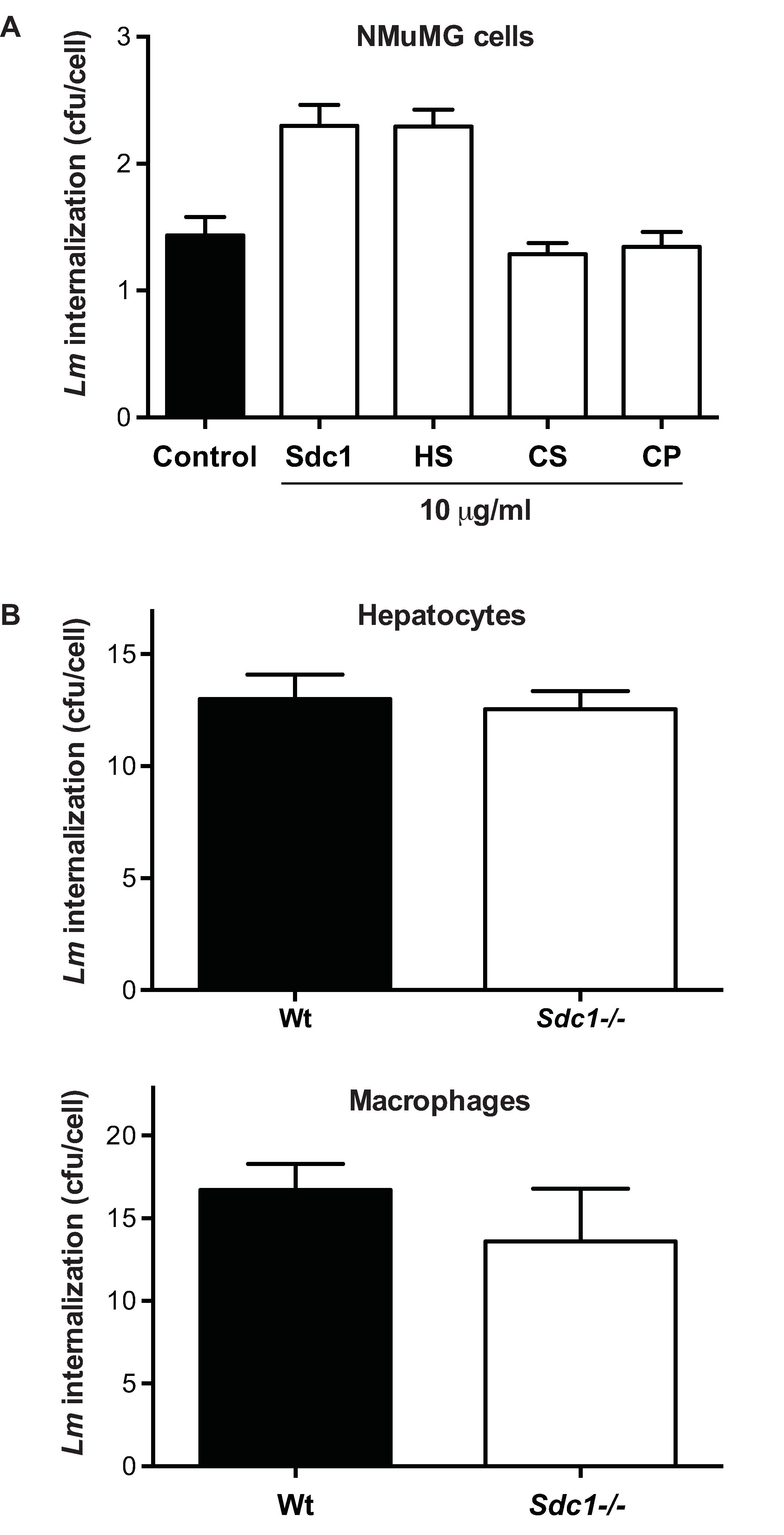

Supplement: S4 Fig — A) Confluent NMuMG cells in 96 well plates were incubated with 4x10^3^ cfu of Lm EGDe (MOI = 0.1) for 2 h at 37°C in the absence or presence of 10 μg/ml purified Sdc1 ectodomain, HS, CS, or core protein (CP). Internalized bacteria were quantified by incubating with 100 μg/ml gentamycin for 30 min to kill extracellular bacteria, washing with PBS, lysing in BHI containing 0.1% Triton X-100, plating out serial dilutions onto BHI agar plates, and counting Lm colonies (n = 3). B) Primary Wt and Sdc1-/- hepatocytes and macrophages in 96 well plates were incubated with 3x10^3^ Lm for 2 h at 37°C and internalized bacteria were quantified (n = 5). (TIF) [file ppat.1008497.s004.tif]

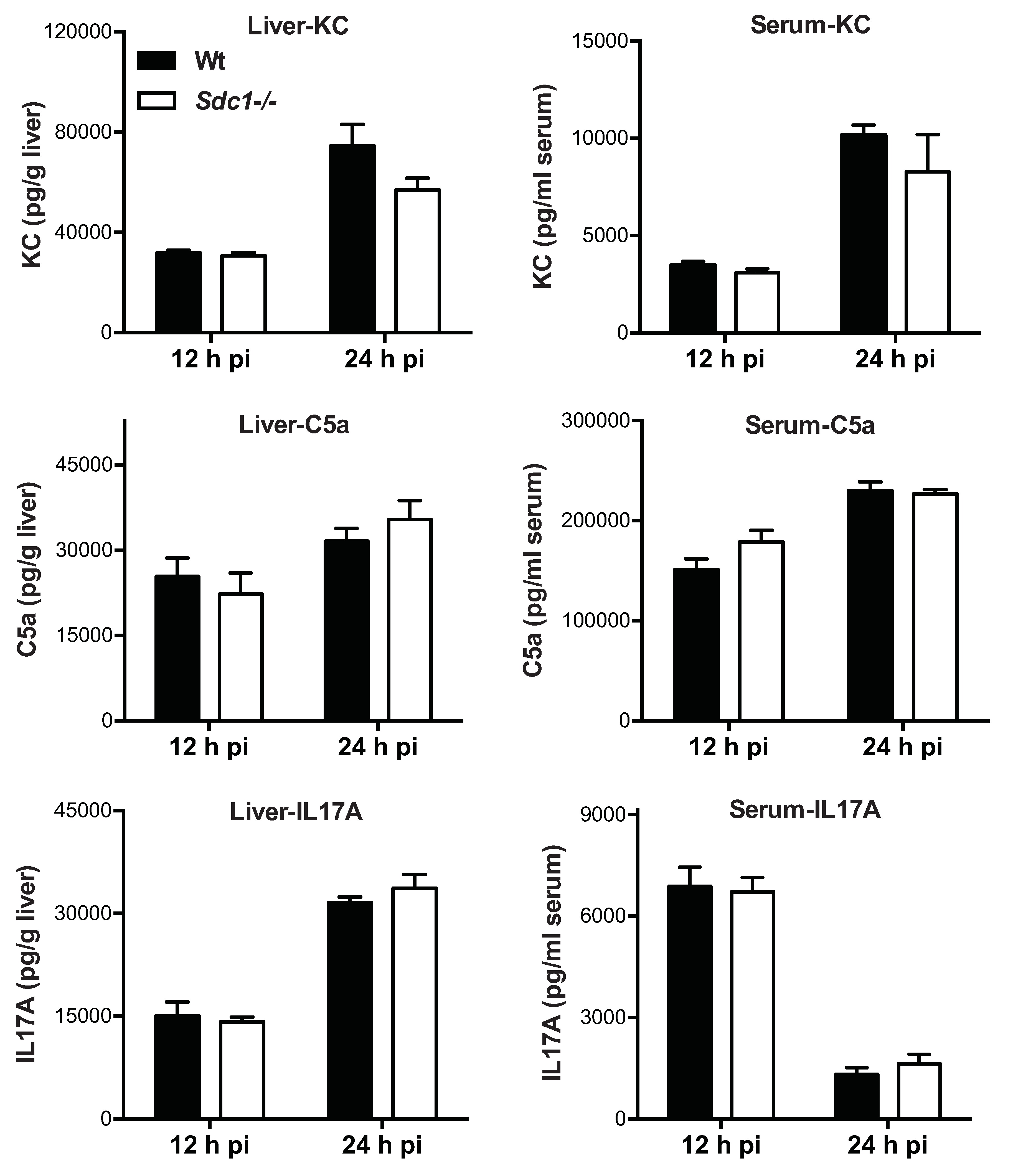

Supplement: S5 Fig — Levels of KC, C5a, and IL-17A in liver homogenates and serum were measured by ELISA (n = 4). (TIF) [file ppat.1008497.s005.tif]

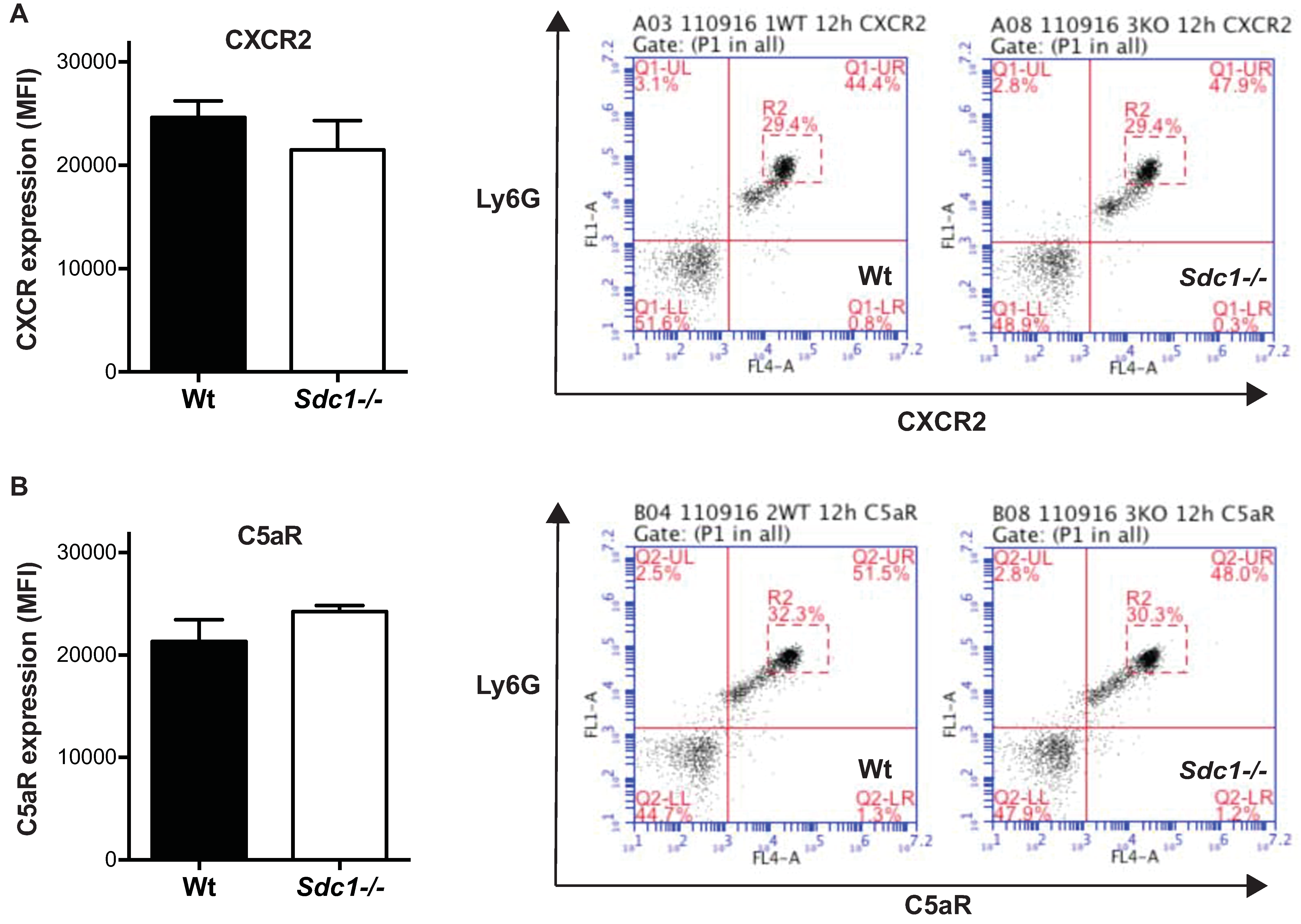

Supplement: S6 Fig — MFI plots (n = 3) and representative dot plots of A) CXCR2 and B) C5aR expression on Ly6G+ neutrophils are shown. (TIF) [file ppat.1008497.s006.tif]

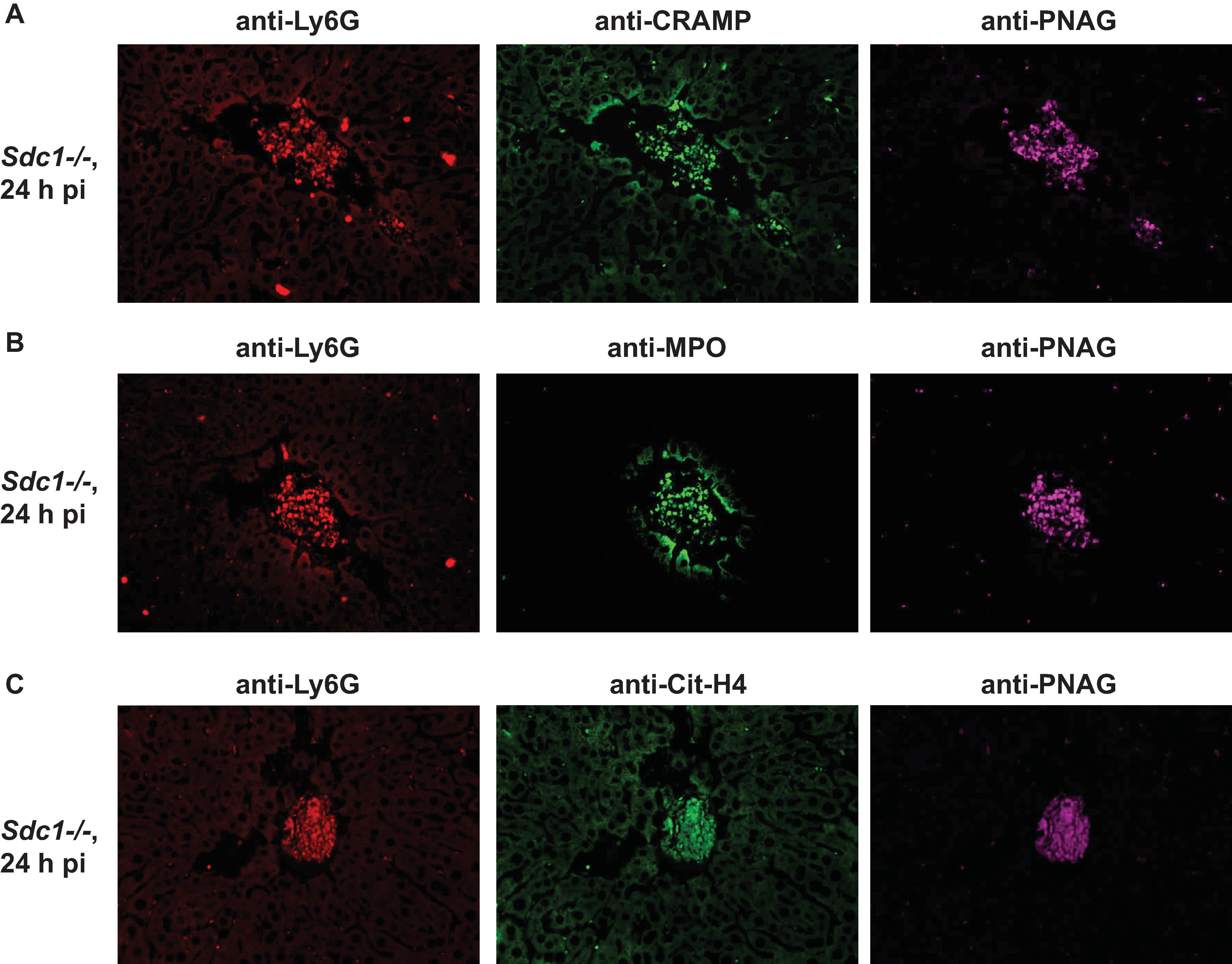

Supplement: S7 Fig — Intravascular inflammatory lesions in Sdc1-/- liver sections (24 h pi) were immunostained for A) neutrophils (anti-Ly6G), CRAMP, and Lm (anti-PNAG), B) neutrophils, MPO, and Lm, or C) neutrophils, citrullinated histone H4, and Lm (original magnification, x200). (TIF) [file ppat.1008497.s007.tif]

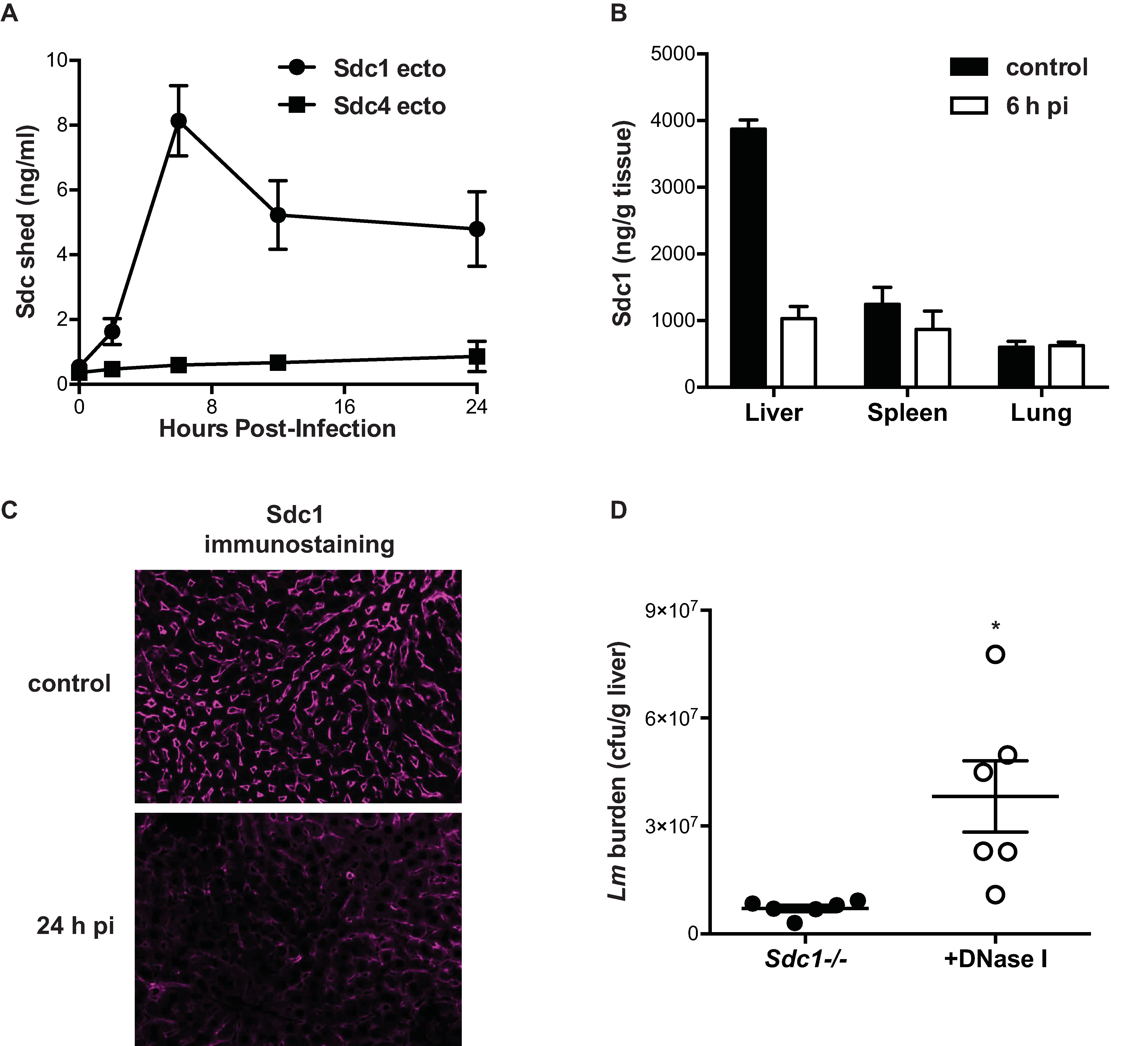

Supplement: S8 Fig — B) Sdc1 levels in liver, spleen, and lung urea extracts were measured before (control) and at 6 h pi (n = 3). C) Liver sections of Wt mice before (control) and at 24 h pi were immunostained for Sdc1 (original magnification, x200). D) Sdc1-/- mice were infected i.v. with 4.5x10^5^ cfu of Lm and injected with PBS or 500 U/mouse DNase I at 12 h pi and the liver bacterial burden was determined at 24 h pi (n = 6, *p<0.05). (TIF) [file ppat.1008497.s008.tif]

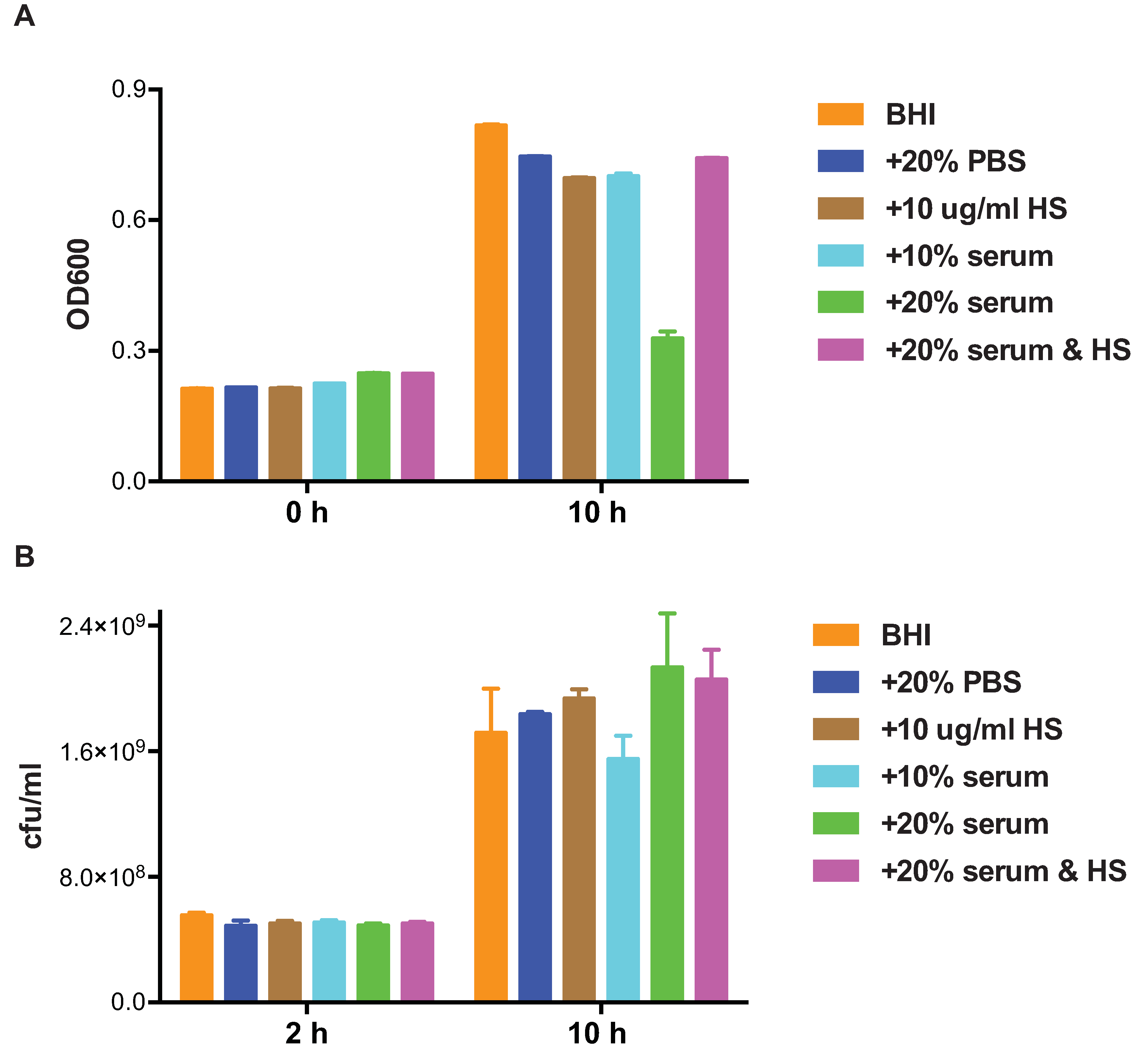

Supplement: S9 Fig — A) Bacterial aggregation was assessed by measuring turbidity at OD600nm (n = 3). B) Bacterial growth was measured by plating serial dilutions at 2 h and 10 h and counting Lm colonies (n = 3). (TIF) [file ppat.1008497.s009.tif]
